# Fabrication of Gelatin Nanofibers by Electrospinning—Mixture of Gelatin and Polyvinyl Alcohol

**DOI:** 10.3390/polym14132610

**Published:** 2022-06-27

**Authors:** Hsiu Yu Chi, Nai Yun Chang, Chuan Li, Vincent Chan, Jang Hsin Hsieh, Ya-Hui Tsai, Tingchao Lin

**Affiliations:** 1 Division of Cardiovascular Surgery, Department of Surgery, Taoyuan Armed Forces General Hospital, Taoyuan 32551, Taiwan; eustonpluto@gmail.com; 2 Department of Biomedical Engineering, National Yang Ming Chiao Tung University, Taipei 11221, Taiwan; abcluluchang@gmail.com; 3 Department of Mechanical Engineering, National Central University, Jhongli, Taoyuan 32001, Taiwan; 4 Department of Biomedical Engineering, College of Engineering, Khalifa University, Abu Dhabi P.O. Box 127788, United Arab Emirates; 5 Center for Plasma and Thin Film Technologies, Ming Chi University of Technology, New Taipei City 24301, Taiwan; 6 Department of Surgery, Far Eastern Memorial Hospital, Banqiao, New Taipei City 22060, Taiwan; yahuitsi@gmail.com; 7 Division of Cardiovascular Surgery, Heart Center, Cheng Hsin General Hospital, Taipei 11246, Taiwan; tingchaolin@gmail.com; 8 Institute of Emergency and Critical Care Medicine, National Yang Ming Chiao Tung University, Taipei 11221, Taiwan

**Keywords:** gelatin, polyvinyl alcohol, electrospinning, spin coating, Fourier-transform infrared spectrometer, water contact angle

## Abstract

Gelatin, one of the most abundant, naturally derived biomacromolecules from collagen, is widely applicable in food additives, cosmetic ingredients, drug formulation, and wound dressing based on their non-toxicity and biodegradability. In parallel, polyvinyl alcohol (PVA), a synthetic polymer, has been commonly applied as a thickening agent for coating processes in aqueous systems and a major component in healthcare products for cartilage replacements, eye lubrication, and contact lenses. In this study, a new type of mixed hydrogel nanofiber was fabricated from gelatin and polyvinyl alcohol by electrospinning under a feasible range of polymer compositions. To determine the optimal composition of gelatin and polyvinyl alcohol in nanofiber fabrication, several key physicochemical properties of mixed polymer solutions such as viscosity, surface tension, pH, and electrical conductance were thoroughly characterized by a viscometer, surface tensiometer, water analyzer, and carbon electron probe. Moreover, the molecular structures of polymeric chains within mixed hydrogel nanofibers were investigated with Fourier-transform infrared spectroscopy. The morphologies and surface elemental compositions of the mixed hydrogel nanofibers were examined by the scanning electron microscope and energy-dispersive X-ray spectroscopy, respectively. The measurement of water contact angles was performed for measuring the hydrophilicity of nanofiber surfaces. Most importantly, the potential cytotoxicity of the electrospun nanofibers was evaluated by the in vitro culture of 3T3 fibroblasts. Through our extensive study, it was found that a PVA-rich solution (a volumetric ratio of gelatin/polyvinyl alcohol <1) would be superior for the efficient production of mixed hydrogel nanofibers by electrospinning techniques. This result is due to the appropriate balance between the higher viscosity (~420–~4300 10^−2^ poise) and slightly lower surface tension (~35.12–~32.68 mN/m^2^) of the mixed polymer solution. The regression on the viscosity data also found a good fit by the Lederer–Rougier’s model for a binary mixture. For the hydrophilicity of nanofibers, the numerical analysis estimates that the value of interfacial energy for the water contact on nanofibers is around ~−0.028 to ~−0.059 J/m^2^.

## 1. Introduction

Among recently developed biomedical materials, polymeric electrospun nanofibers have emerged as one of the most promising platforms for building multifunctional scaffolds in wound treatment. In general, modern wound dressings must embrace several key functions including the reduction of bleeding, acceleration of blood coagulation, protection of wounded tissues from infection, absorption of exudate, prevention of maceration, stimulation of tissue regeneration, and alleviation of pain with analgesic or antibacterial medications [1,2,3,4,5]. To date, innovative wound dressings rendered with superior bioactivities and engineered precisions can be fabricated into various formats such as porous scaffolds or implants with the use of additive manufacturing for the treatments of various diseases [6,7,8]. In particular, the multicomponent electrospun nanofibers composed of polymeric complex coacervates are superbly fit for the development of novel wound dressing based on their large surface-area-to-volume ratio, high water retention, and unmatched versatility in material selections.

The biomedical applications of hydrogel nanofibers have gone beyond wound dressing. With assorted compositions, ingenious designs, and economical fabrications, hydrogel nanofibers have been used for drug delivery, tissue engineering, and prosthetic products depending on material properties and functions. For example, poly(ethylene-co-vinyl alcohol) (EVOH) in a nanofiber mesh is capable of selectively adsorbing uremic toxins such as creatinine for potential filtration in kidney dialysis [9]. Poly (ethylene oxide) (PEO) and PVA nanofibers, either in the form of blend or core-shell, can encapsulate drugs with a controlled release feature [10]. The superabsorbent textiles of polyvinylpyrrolidone (PVP) and polyacrylic acid (PAA) can be made for gauze sponges [11]. Slowly degradable polyphenols can be coated on orthopedic implants to promote bone regeneration. When added to nanofiber scaffolds, polyphenols even showed some antioxidant effects [12]. Silk fibroin nanofibers, a complicated natural hydrogel, are a respectable constituent in artificial skin, considering their exceptional biocompatibility [13]. 

The two most critical requirements of advanced wound dressing are biocompatibility and nontoxicity, regardless of the site of implementation, and they are fulfilled by various types of electrospun nanofibers. Specifically, polymeric hydrogels derived from naturally occurring biomacromolecules from animals or plants have demonstrated exceptional biocompatibility and ideal biodegradability under a range of in vivo conditions [14,15,16,17]. Among various natural hydrogels, gelatin is perhaps the most abundant of proteins derived from collagen commonly found in the connective tissues of animals. Gelatin is generally produced from the irreversible hydrolysis and denaturation of collagen boiling with the use of acids (type A) or alkalis (type B) [18,19,20,21,22]. The exact compositions of gelatin in terms of amino acid sequences vary based on the source and extraction methods. In general, gelatin isolated from various types of animal tissues is translucent under visualization at room temperature and rich in glycine, proline, hydroxyproline, alanine, etc. [23,24,25,26,27]. After gelatin synthesis, various purification steps including filtration, evaporation, refining, recovery, drying, or grinding are applied to form the final products [18,19,20,21,22]. Gelatin is also used as an additive in food, cosmetics, or medicine [28,29,30,31,32].

Alternatively, hydrogels composed of synthetic polymers such as poly(vinyl alcohol) (PVA, [CH_2_CH(OH)]_n_) emerge as a simple and low-cost thickening agent that has been extensively applied in the manufacturing of several biomedical products such as contact lenses, stents, and cartilage implants [33,34,35,36,37]. Similar to gelatin, PVA is a water-soluble and biocompatible polymer, serving as a good adhesive for bonding insulators such as wood and paper. Moreover, PVA is produced in the form of colorless and odorless powders, which readily dissolve in water to form a viscous liquid [37]. Interestingly, the gelation of a PVA solution enables the combination of PVA with other molecular constituents such as borate (boron–oxygen compounds) to fabricate novel hydrogel with enhanced mechanical properties for various biomedical applications under physiological conditions [38].

Although the potential fabrication of gelatin into nanofibers sounds attractive for advanced biomedical applications, a pure gelatin solution is not a feasible choice for direct electrospinning. For instance, the gelation and melting temperatures of gelatin derived from goats are found to be around 25.14–25.23 and 34.09–34.18 °C, respectively. The thermal properties mentioned above lead to the rapid solidification of the gelatin solution into a solid at the operation temperature (23 °C) of conventional electrospinning [39]. At the same time, the low surface charge density of gelatin prohibits effective nanofiber formation during electrospinning. Most importantly, the highly sensitive, temperature-dependent viscosity of the gelatin solution imposes a significant challenge in the preparation of practically feasible gelatin solutions for electrospinning or spin coating. The shared physiochemical properties between gelatin and PVA as mentioned above have attracted the attention of some researchers for fabricating novel gelatin/PVA composite scaffolds for emerging applications in tissue engineering [10,11,33,34,35,36,37]. To date, the formation of electrospun gelatin/PVA hydrogel nanofibers has not been systematically studied against different experimental conditions. Such an approach of blending between gelatin and PVA will potentially stabilize the nanofiber formation from electrospinning. 

It has been shown that acetic acid at sufficiently high concentrations can reduce the molecular weight of gelatin through hydrolysis of a fraction of the peptide bonds along the polymeric backbone of gelatin [19,20,21,22]. Specifically, the hydrolysis effectively prevents the rapid solidification of the gelatin solution at room temperature and enables the effective mixing of gelatin with PVA in the liquid phase. Therefore, the preparation of a gelatin/PVA polymer solution that is applicable for both spin coating and electrospinning should be achievable by applying a certain concentration of acetic acids during the mixing of the two polymers.

In this study, we fabricated gelatin/PVA nanofibers by electrospinning on top of a hydrogel film of identical compositions produced by spin coating. The structure, morphology, and compositions of the gelatin/PVA nanofibers were systematically examined against a range of physicochemical properties of polymer solution mixtures. In particular, the viscosity, surface tension, electrical conductance, and pH of gelatin/PVA polymer solutions used in the fabrication of hydrogel nanofibers and films were examined by a viscometer, surface tensiometer, digital carbon electrode, and digital electrochemical probe, respectively. Afterward, a scanning electron microscope (SEM) was applied to probe the surface morphology, while a Fourier-transform infrared spectrometer (FTIR) was applied to probe the chemical structures, and water contact angle measurement was used to evaluate the hydrophilicity of gelatin/PVA nanofibers prepared from different polymer solution mixtures. To test the cytotoxicity of gelatin/PVA nanofibrous film, MTT assays of cultured 3T3 fibroblasts for determining the live/dead cell ratio were performed. Overall, the interrelation between polymer solution properties, hydrogel nanofiber structures, and cellular responses was elucidated herein for enabling the future development of highly engineered gelatin/PVA nanofibers in specific applications.

## 2. Experimental Procedure

**Figure 1:**
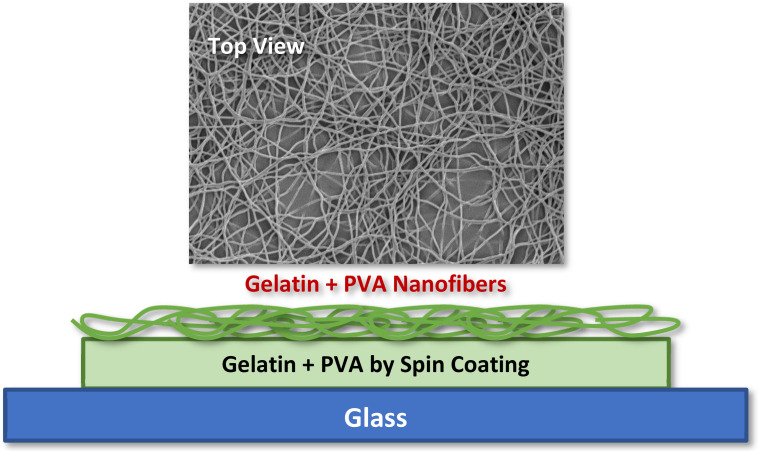
Morphology of electrospun gelatin/PVA mixed nanofibers on top of a film made of identical material.

The design of the gelatin/PVA nanofiber-film pack is depicted in Figure 1 where both the layer film and nanofibers are made of mixed gelatin (12 wt. % in DI water) with acetic acids (10 vol. % in DI water), and/or PVA (12 wt. % in DI water) is formed by spin coating and electrospinning, respectively. The main purpose of adding acetic acids to gelatin is to prevent the solidification of gelatin at room temperature. There is no such need for pure PVA solution since the pure PVA is already in the liquid state at room temperature. For all testing and material characterizations, we place the whole pack on a glass substrate for easy handling and observations of cell culture.

### 2.1. Substrate and Materials

Corning glass (Corning 1737) was cut into 10 × 10 mm^2^ pieces by tungsten cutter and then ultrasonically cleaned in KOH (85%), acetone (99.9%), DI water, and alcohol (90%) alternatively. Each cleaning took around 10 min. After clean-up, the cut glass was blown dry by nitrogen. For the cell culture, all deposited film samples were radiated by UV light for at least 15 min and then placed in a 24-well dish for all biological tests.

Purchased gelatin (2013-0311-076, major amino acids: AGPRGEOGPG, ~20.6 cps, Emperor Chemical Co., Ltd., Taipei, Taiwan) in the form of white to light yellow powders was dissolved in DI water and acetic acid (2013-0208-022, 99.5%, Emperor Chemical Co., Ltd., Taipei, Taiwan) then continuously stirred for 1–2 h ~70 °C before being used for spin coating or electrospinning. The concentration of gelatin and acetic acids is listed in Table 1 and Table 2.

PVA (~120,000–132,000 g/mol, ~36–42 cps, 2013-0401-084, Emperor Chemical Co., Ltd., Taipei, Taiwan) in white powders was dissolved in DI water and then continuously stirred for 1–2 h at room temperature before being used for spin coating or electrospinning. The concentration of PVA is also listed in Table 1 and Table 2.

In this study, we tested gelatin and PVA solutions mixed at different volumetric ratios prepared for films by spin coating, and nanofibers by electrospinning. This information is crucial for the production of such nanofibers at large scales.

### 2.2. Spin Coating

The gelatin/PVA layer film is formed by spin coating (SWIENCO SP-02, Power Assist Instrument Scientific Corp. Taoyuan, Taiwan) using mixtures of gelatin/PVA solution. The optimized rotation speed was set to 3000 rpm from different films. All coating processes took 1 min to allow the solution to spread large enough to cover the glass substrate. After coating, we let the sample rest calmly for less than 30 min before electrically spinning the nanofibers on top.

### 2.3. Electrospinning

The electrospinning of gelatin/PVA was carried by a commercial system (FES-COE, Falco, Taiwan) where the system consists of a syringe pump, a power supply, and a horizontally movable platform (collector) to carry the substrate.

The dissolved gelatin/PVA solution was first drawn into a syringe tube before ejecting onto a substrate. Parameters for the operation of the electrospinning system are listed in Table 1 and Table 2. Note that particularly in the electrospinning, the distances between the needle and substrate and voltage are all adjusted by trial and error in a preliminary study to have an optimal result of deposited gelatin/PVA with uniform thickness. We skip details for the conciseness of this report. Moreover, the formation of nanofibers is mostly unaffected by the compositions of gelatin/PVA using the current equipment. The dimension (diameter) of nanofibers is controlled by both voltage and syringe pumping speed. In general, the higher the applied voltage or pumping speed, the thinner the fibers that are produced. However, excessively high voltage can induce arching between the substrate and needle, which leads to the breakup of a jet into droplets. Parameters in Table 1 and Table 2 were chosen by many rounds of trials to assure the successive reproduction of gelatin/PVA nanofibers.

### 2.4. Characterization

#### 2.4.1. Viscosity of Polymer Solution

The viscosity of gelatin and PVA mixed solution is measured by a viscometer (DV2T, Brookfield AMETEK, Middleborough, MA, USA). The measurement was carried out at room temperature with a spindle (SC4-27D). The rotational velocity is set at 20 rpm for generating enough torque. It is suggested by the manufacturer to have at least 10% of the maximum torque to obtain accurate measures for viscosity. The measured viscosity is automatically recorded by the system every 15 s. An accurate measure typically takes about 2–5 min until the fluid flow reaches a steady state. Each sample solution was repeatedly measured 3 times, and the average was calculated.

#### 2.4.2. Electrical Conductivity and pH Value of Polymer Solution

The electrical conductance and pH of gelatin/PVA solutions were measured by a multiple-purpose water analyzer (WA-2017SD-Water Analysis, Lutron, Taiwan). For the pH measurement, an electrochemical probe (PE-03) is connected to a multiple-purpose datalogger for detection, whereas for the measurement of electrical conductance, a carbon electrode (CDPB-03) is instead used with a connection to the same datalogger.

For better accuracy, three measurements were taken for both electrical conductance and pH value in each sample solution to have averaged values.

#### 2.4.3. Surface Tension of Polymer Solution

The surface tension of gelatin and PVA mixed solutions is measured by a digital surface tensiometer (BZY-IV, Hengping Instrument Ltd., Shanghai, China). The measurement follows the Wilhelmy plate method. A rectangular iridium–platinum plate of dimensions 23.8 mm (w) × 10 mm (h) × 0.43 cm (t) is first dipped into the liquid and then gradually pulled back by a thin wire to its original position before dipping. The whole process is precisely controlled by a height-adjustable table that carries a glass beaker of liquid while a stationary fixture hangs the plate still. During the pulling, liquid lamella snaps on the plate, and the force balance between the weight of lamella and the liquid/plate interfacial tension leads to the Wilhelmy equation:

(1) $$\gamma_{LP}=\frac{F}{l_{wet}\cos\theta }$$

where *γ_LP_* is interfacial tension (energy) between the liquid and plate, *F* is the measured hanging force through the wire, *l_wet_* is the wetted perimeter (2 × (width + thickness)), and *θ* is the contact angle between the liquid and the plate. In practice, the difficulty of measuring contact angle is either replaced by values from the literature if the liquid is well known or assuming complete wetting (*θ* = 0) instead. In this study, complete wetting is assumed because both dipping and pulling are slow (2–3 min), and we added a period of waiting after submerging.

#### 2.4.4. Surface Morphology of Layer Film and Nanofibers

The morphology of electrospun gelatin/PVA fibers was imaged and estimated by scanning electron microscope (SEM, S-3400N, Hitachi, Japan). For SEM, the voltage of the accelerated electron beam is set at 15 kV, the working distance is 10,300 μm, the emission current is 88,000 nA, and the magnification is chosen to be 3000 for the best resolution.

#### 2.4.5. Chemical Element Analysis of Layer Film and Nanofibers

The average elemental compositions of electrospun gelatin/PVA fibers under SEM inspection were measured by energy-dispersive X-ray spectroscopy (EDS, EDS, Bruker Nano, XFlash Detector 5010, German) using a 15 kV electron beam; the counting per second (cps) is between 2000–3000, and scanning area is around 1500 × 1125 pixels. The magnification was chosen to be 3000 too. The selected chemical elements subjected to analysis are C, O, and N from the radiation of the Kα series.

#### 2.4.6. Molecular Structure of Layer Film and Nanofibers

To determine the vibrational modes of molecular bonding in the deposited gelatin/PVA layer films, Fourier-transform infrared spectrometer (FTIR, V-770, Jasco USA, equipped with single monochromators and dual detectors) was used to detect the infrared absorption in the range of 650–4000 cm^−1^ with a resolution of 1 cm^−1^ in the reflection mode. An uncoated glass substrate was first detected, and its spectrum is used as a reference to differentiate the spectrum from coated samples. During measurement, the detector is in line with the sample at its backside. The spectrum was numerically analyzed by Fityk 0.9.8 using Gaussian fitting to identify possible absorption peaks for different vibrational modes of various chemical bonds. Major absorption peaks of optical emission relevant to gelatin/PVA films are listed in Table A1 in Appendix B for later discussion [40,41,42,43,44,45,46,47,48,49,50].

#### 2.4.7. Contact Angle of Layer Film and Nanofibers

The contact angles of electrospun gelatin/PVA fiber films were measured from the image of water droplets (1 mL) on samples and shot by a CCD camera (Watec, WAT-902B, Watec Corp., Tsuruoka, Yamagata, Japan). Images were first uploaded to the “Online Protractor” (https://www.ginifab.com/feeds/angle_measurement/, accessed on 1 March 2022), and then the contact angle of drops on substrates could be measured.

## 3. In Vitro Cell Culture

### 3.1. Cell Preparation

3T3 fibroblasts purchased from (ATCC, CCL-92^TM^, 3T3-Swiss albino, Research Center, Hsinchu, Taiwan) are ready for the experiment. The cells, stored in tubes and kept in a −20 °C freezer, were first bathed and shaken in 37 °C water for about 30 min. After completely warming up, cells are pipetted into a culture medium (Dulbecco’s Modified Eagle Medium/High Glucose powder, Gibco^®^, Thermo Fisher Scientific, Waltham, MA, USA) inside a 6 cm diameter Petri dish. Using our own established protocol, 3T3 fibroblasts were cultured in an incubator (2424IR, ShelLab-Sheldon Manufacturing Inc., Cornelius, OR, USA) under a controlled environment, i.e., 37 °C, 5% CO_2_, and 95% relative humidity, for 2 to 3 days before they were trypsinized and pipetted into tubes. This suspension was then centrifuged for 5 min at 1500 rpm. After centrifugation, cells in the sediment mixed with culture medium were pipetted out and dropped into a 6- or 24-well plate for further tests.

Note that for the cell culture, all fiber film samples were radiated by UV light for at least 30 min and then placed in a 24-well dish before biological tests.

### 3.2. Cell Viability Test

To access the cell viability of 3T3 by in vitro culture, the culture processes were explained as follows. We first immersed electrospun gelatin/PVA samples in 500 μL of Dulbecco’s Modified Eagle Medium (DMEM) inside a 24-well plate. Then, 3T3 fibroblasts (25,000 cells/well) were directly seeded on each well. After 48 h, images of seeded cells were taken under an optical microscope (50×). After imaging, the DMEM was abstracted and proceeded to the MTT assay.

Another seeding process is to immerse fiber film samples in the 24-well plate and seed the 3T3 fibroblasts (25,000 cells/well) directly on the samples. After 48 h, optical images of seeded cells on samples were taken, and the DMEM was abstracted for the MTT assay.

### 3.3. MTT Assay

The viability of cells is evaluated by MTT assay. MTT is a yellowish and water-soluble tetrazolium salt (3-[4,5-dimethylthiazol-2-yl]-2,5-diphenyltetrazolium-bromide). If there is nicotinamide adenine dinucleotide (generated by active metabolism in cells, its oxidized and reduced forms shortened as NAD^+^ and NADH, respectively) present in the solution, MTT can then be reduced to an insoluble purple formazan. This insoluble formazan shall deposit on the cell surface and can be dissolved in isopropanol or other organic solvents. The dissolved solution has signature optical absorbing peaks at 550 and 650 nm identifiable by a spectrometer (Epoch 2, Microplate Spectrophotometer, BioTek Instruments Inc. Santa Clara, CA, USA), the intensities of which are proportional to the concentration of formazan. As MTT is found in all living cells, its concentration can be used as a marker to quantify the number of viable cells. The intensity of absorption peaks is usually expressed as optical density (OD), which is defined as

(2) $${\mathrm{OD}=\log}_{10}\left(\frac{\mathrm{incident}\;\mathrm{light}\;\mathrm{intensity}}{\mathrm{transmitted}\;\mathrm{light}\;\mathrm{intensity}}\right)$$

The steps of the MTT assay are described as follows. The abstracted DMEM was mixed with 200 μL MTT (0.5 mg/mL in 1X PBS) and pipetted into a 24-well plate. The plate was kept in the incubator for 2–4 h and then examined by an optical microscope. If crystalline formazans (filaments) were found in cells, another 600 μL dimethyl sulfoxide (DMSO, (CH_3_)_2_SO) was then added to each well to dissolve formazans. After around 5 min, 200 μL of DMSO-added culture media was abstracted from each well and distributed into a 96-well plate. This plate was sent to the spectrometer for measuring the optical absorption by liquid at 550 nm and 650 nm.

## 4. Results

### 4.1. Characterizations for Polymer Solution

#### 4.1.1. Viscosity

**Figure 2:**
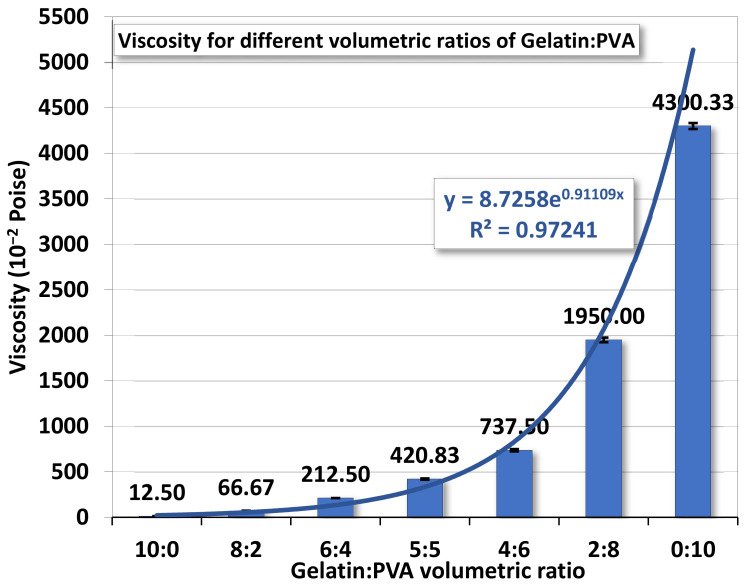
The averaged viscosity of gelatin/PVA mixed solution.

Whether mutually miscible gelatin and PVA solutions have an impact on the polymeric solution prepared for electrospinning remains to be investigated. Figure 2 demonstrates the variation of viscosity according to different gelatin/PVA volumetric concentrations. The variation seems to follow an exponential function by numerical fitting with a coefficient of determination of ~0.9723.

The variation of viscosity is much like a continuous function without a first- or second-order phase change. In other words, the mutually miscible gelatin and PVA solution should not involve chemical reactions. For the pure gelatin solution, the low viscosity, 12.5 centipoises, reflects its high solubility in DI water while the pure PVA (12 wt. %) having a viscosity reaching 4300.33 centipoises implies a much higher resistance to water dissolution. This phenomenon is largely attributed to the difference in the molecular structures of the two polymers. The gelatin has a long chain of amino acids vulnerable to hydrogen bonding by water molecules, whereas the smaller and more compact secondary alcohol structure 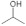 in PVA is better for resisting such water bonding [51].

#### 4.1.2. Electrical Conductance

**Figure 3:**
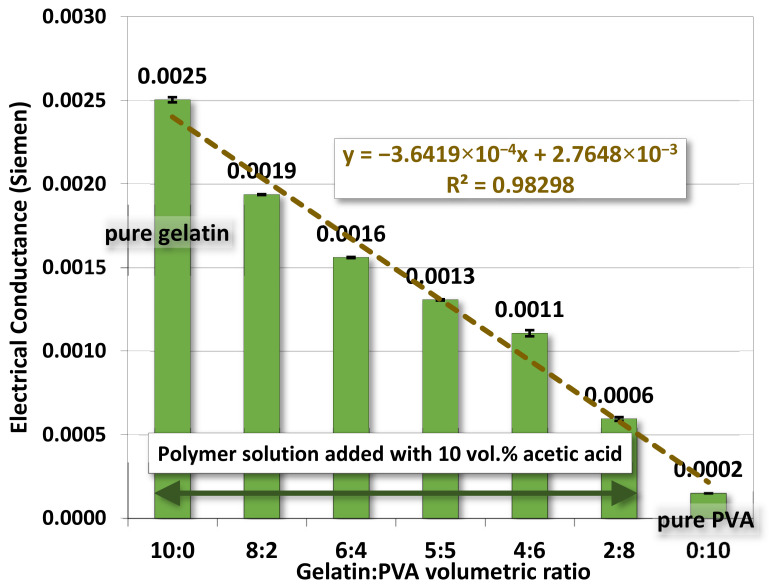
The average electrical conductance of gelatin/PVA mixed solutions.

Figure 3 shows the electrical conductance of a polymer solution against the change of gelatin/PVA volumetric or concentration ratio in the polymer solution mixture. The result showed that the electrical conductivity for the gelatin/PVA solution mixture was lowered against the increase in the relative amount of PVA. For the pure PVA solution (12 wt. %), the electrical conductivity was lowest at 1.5 × 10^−4^ S in the absence of acetic acid among all samples used herein. This was because PVA has a low degree of ionization, even at lower pH [52]. Interestingly, higher electrical conductivity was detected in all gelatin/PVA mixtures compared to that of pure PVA. The result was mainly promoted by the presence of acetic acids used for gelatin dissolution (for all gelatin-containing mixtures), leading to higher ionic strength in DI water [19,20,21,22]. So, even for the solution of gelatin/PVA ratio at 2:8, the solution conductivity, measured to be around 5.9 × 10^−4^ S, is still about four times higher than that of the pure PVA solution.

The variation of electrical conductivity for gelatin/PVA mixed solutions is numerically fitted well by a linear function with the coefficient of determination R^2^ = 0.975. These fitting results provide a guideline for the preparation of polymer solutions in successive studies.

#### 4.1.3. pH Value

**Figure 4:**
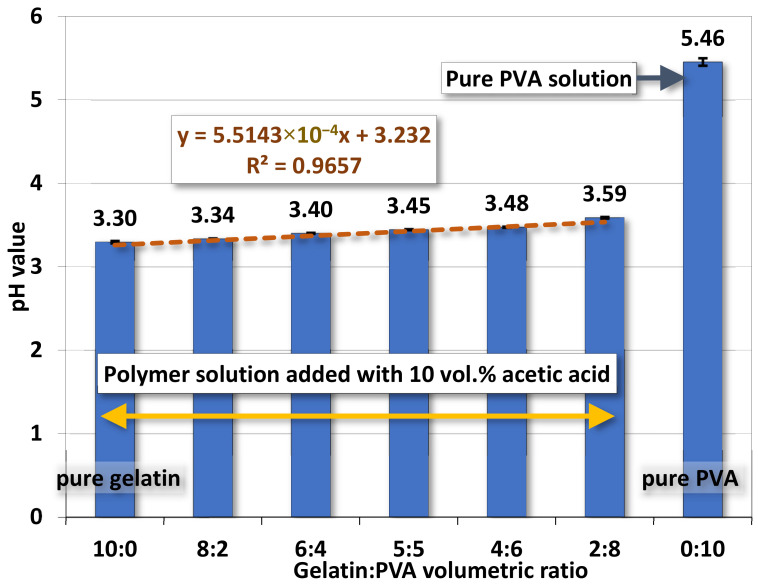
The average pH values of gelatin/PVA mixed solutions.

The variation of pH values for gelatin/PVA mixed solutions, shown in Figure 4, is influenced by the adding acetic acids. For various gelatin/PVA mixed solutions, the pH values increase as the gelatin content is reduced. However, the change in pH value is very minor (3.30 for gelatin/PVA = 10:0 to 3.59 for gelatin/PVA = 2:8) because the whole solution is primarily a mixture of nothing but weak acids - amino acids, acetic acids, and PVA (the pH value of pure PVA without acetic acids is around 5.5). Note that most acetic acids will evaporate out when layer films or nanofibers start to form through solidification in the process of spin coating and electrospinning.

Variations of the pH values are also numerically fitted against the change of gelatin/PVA volume ratio. Similar to the case of electrical conductivity, a linear function is found with a high coefficient of determination R^2^ = 0.966.

#### 4.1.4. Surface Tension

**Figure 5:**
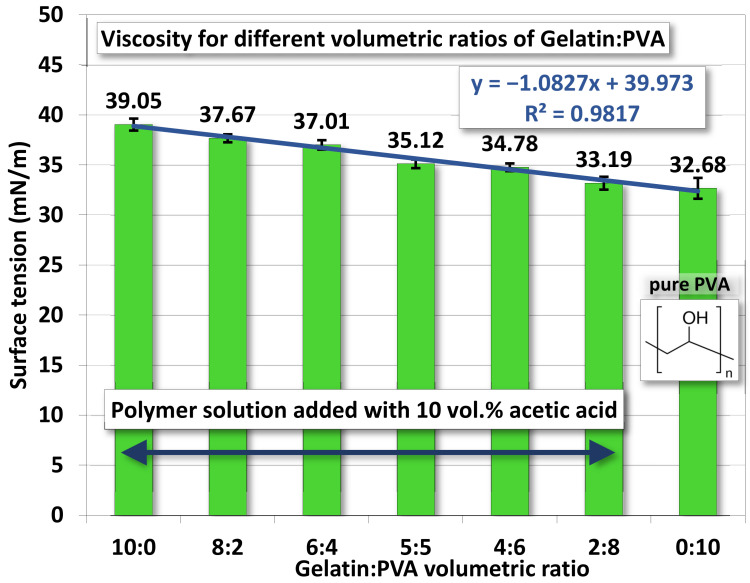
The average surface tension of gelatin/PVA mixed solutions.

The surface tension for gelatin/PVA mixed solutions is shown in Figure 5. Values of surface tension decrease as the PVA concentration increases. A linear polynomial function is found to fit measurements with R^2^ = 0.982. Pure PVA has a higher viscosity, but its surface tension is nevertheless lower than all other mixed solutions. These two opposites combined can be very helpful in the formation of nanofibers by electrospinning, as lower surface tension facilitates the stretching by the electrical field while higher viscosity prevents the disintegration of fibers into droplets (see SEM in the next section).

### 4.2. Characterizations for Layer Films and Nanofibers

#### 4.2.1. Morphology of Nanofibers

**Figure 6:**
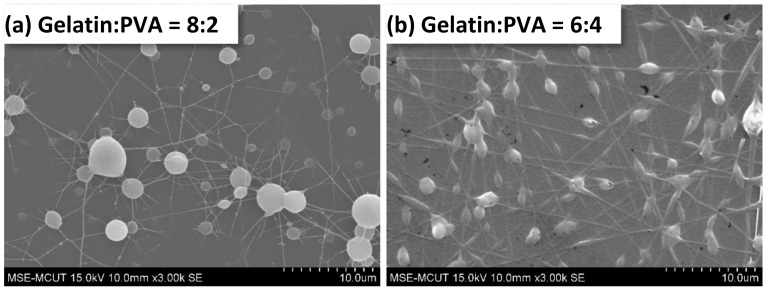

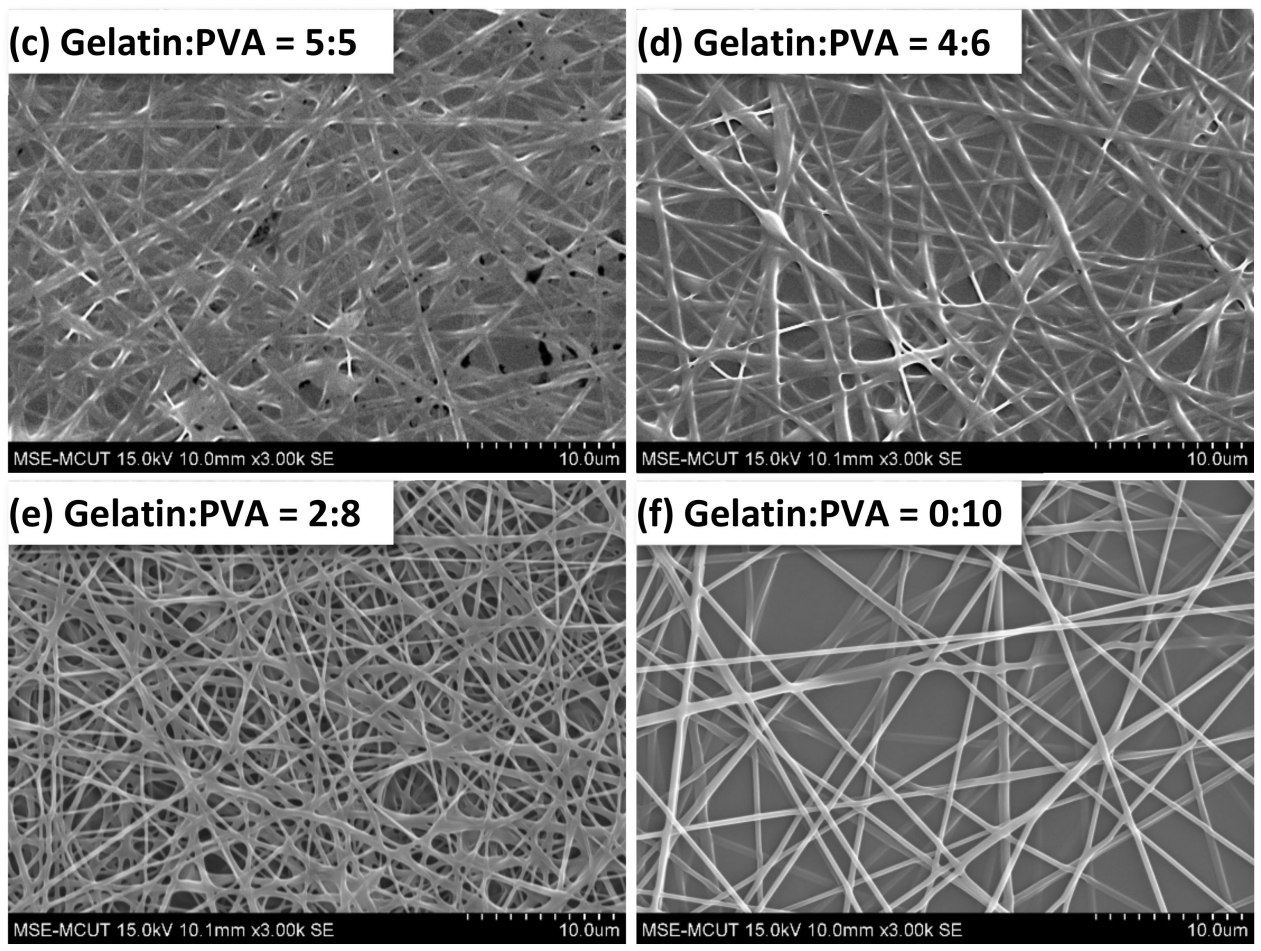
Morphology of electrospun gelatin/PVA mixed nanofibers on top of a film.

The SEM images of gelatin/PVA nanofibers are presented in Figure 6. It shows that no fibers were formed by electrospinning at low PVA concentrations (Figure 6a the volumetric ratio of gelatin/PVA = 8:2). As the content of PVA increases, the nanofibers start to form as shown in Figure 6b (the volumetric ratio of gelatin/PVA changes to 6:4). The mixed gelatin/PVA fibers shown in Figure 6c at a volumetric ratio of 5:5 seem to be mutually miscible; therefore, much thicker fibers are observed. As the PVA concentration becomes a dominant composition, i.e., the volumetric ratio of gelatin to PVA is lower than 5:5, nanofibers become thinner, less overlapping, and tangling (Figure 6d–f). Less overlapping and tangling implies two phenomena: the higher stretching of jets and the easy whipping of nanofibers.

**Figure 7:**
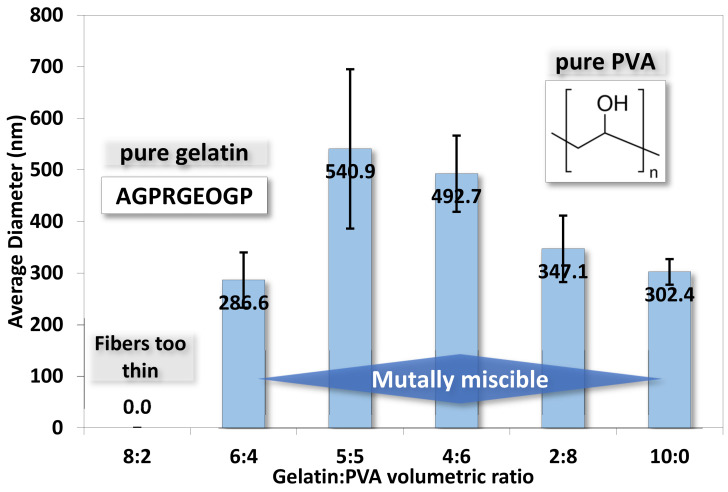
The averaged diameters for electrospun gelatin/PVA mixed nanofibers. (The symbols for amino acids are A: alanine, G: glycine, P: proline, R: arginine, E: glutamate, and O: hydroxyproline).

To quantify the nanofibers’ dimension, we randomly selected fibers from each SEM image and measured their diameters. The average diameters of fibers in different samples are shown in Figure 7. The range of average diameters of fibers falls between 287 and 541 nm, but the diameters of pure 50:50 gelatin/PVA fibers are larger than other samples. The formation of larger diameters can be attributed to the thinning of jets and mutually miscible gelatin and PVA solutions, as observed in the SEM images.

#### 4.2.2. EDS

**Figure 8:**
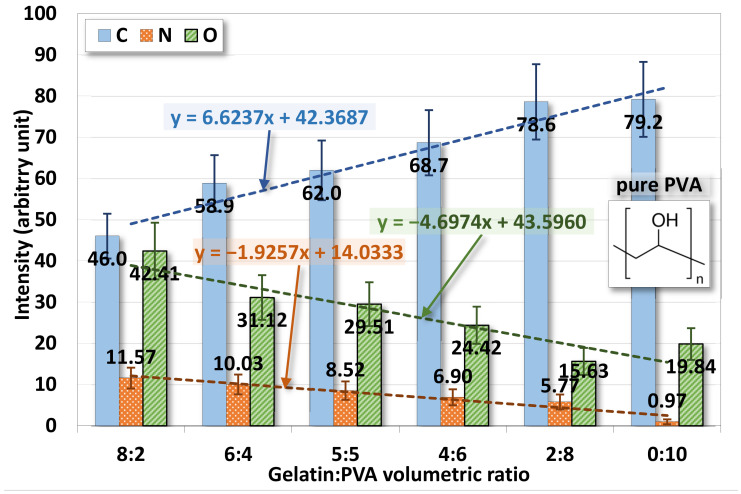
Variations of surface chemical elements from the EDS of electrospun gelatin/PVA mixed nanofibers.

The chemical element analysis on the surface of gelatin/PVA fiber films is shown in Figure 8 where three elements, carbon, oxygen, and nitrogen, are identified. Note that only a trace of 0.97 at. % is found in pure PVA because no nitrogen exists in PVA. The content of nitrogen in gelatin/PVA fiber films also varies positively according to the increase of gelatin.

EDS confirms that the chemical compositions of deposited fiber films are directly derived from the polymer solutions prepared for the electrospinning and spin coating. The mixing of gelatin and PVA in DI water is only physical but not chemical.

#### 4.2.3. FTIR

**Figure 9:**
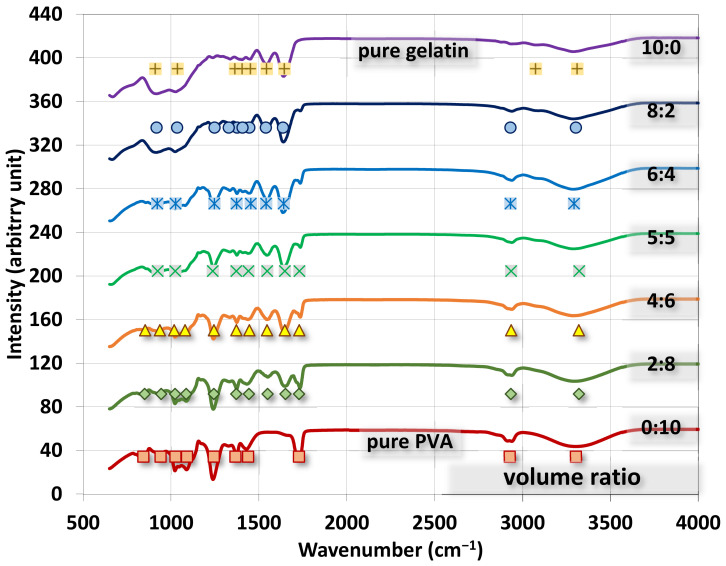
FTIR spectra for electrospun gelatin/PVA nanofiber films with different volumetric mixing ratios.

The FTIR spectra for gelatin/PVA fiber films are shown in Figure 9 where all spectra are presented in the range of 650–4000 cm^−1^ with marks for absorption peaks by Gaussian fitting. We also present spectra for pure gelatin alongside the mixed polymers for benchmark comparison.

Major FTIR peaks by Gaussian fitting for PVA are found to be a C-O stretch at ~1243.3 cm^−1^, C=O at ~1728.3 cm^−1^, O-C-O in ester groups at ~1090.9 cm^−1^, C-O stretch in acetyl groups at ~1025.5 cm^−1^, O-H stretch at ~3304.1 cm^−1^, CH_3_ bending at ~1367 cm^−1^, and CH_2_ bending at ~1439 cm^−1^. For gelatin, major peaks are found to be a C=O stretch at ~1645.2 cm^−1^, amide III at ~1036.9 cm^−1^, δ-NH or C-N stretch at ~1543.5 cm^−1^, -CO-NH-moiety at ~910.1 cm^−1^, C-O-C stretch at ~1363.4 cm^−1^, NH or OH stretch at ~3311.4 cm^−1^, and O-C-O symmetric stretch at ~1451.9 cm^−1^ [40,41,42,43,44,45,46,47,48,49,50]. The rest of the modes are listed in Table A1 for readers’ reference.

As the volumetric ratio of gelatin to PVA changes from pure gelatin (10:0) to pure PVA (0:10), the absorption peaks also change from gelatin’s features to PVA’s signatures. The most observable changes of peaks in Figure 9 due to mixing are the vanish peak at (2927 cm^−1^ of CH sp3 in pure PVA) → pure gelatin; (1728 cm^−1^ of C=O in pure PVA) → (1645 cm^−1^ of C=O stretch in pure gelatin); the vanish peak at (1243.3 cm^−1^ of C-O stretch in pure PVA) → pure gelatin; the vanishing twin peaks (1025.5 cm^−1^ of C-O stretch and C-O-C stretch of 1090 cm^−1^ in pure PVA) → pure gelatin.

#### 4.2.4. Contact Angle

**Figure 10:**
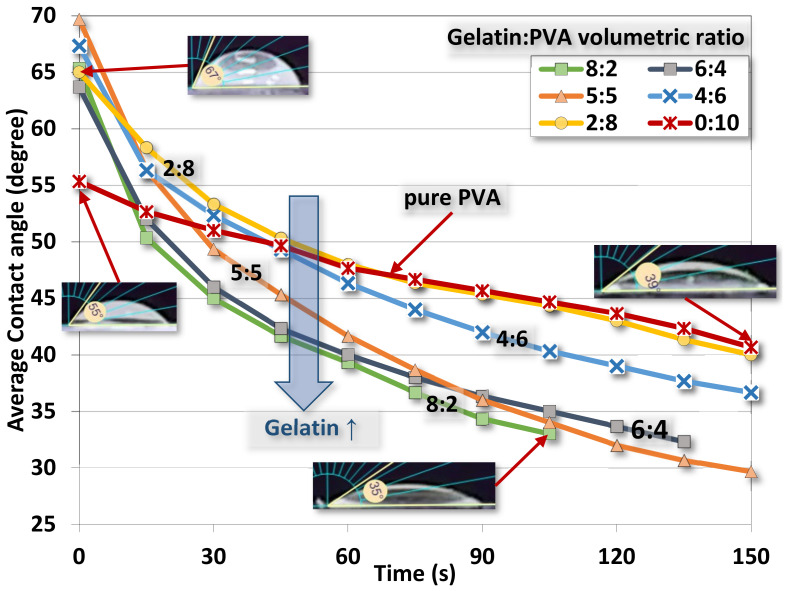
Water contact angle as a function of time for electrospun gelatin/PVA nanofiber films with different volumetric mixing ratios.

The averaged contact angles of gelatin/PVA fiber films are shown in Figure 10 where the time variation of the contact angle declines as the water drop is gradually absorbed into the fibers and films. There is a trend of such decreases corresponding to different compositions. When gelatin/PVA = 8:2, the contact angle decreases from 65° to 33° in about 105 s, while the opposite concentration, gelatin/PVA = 2:8, has the contact angle reduced from 65° to 40° in 150 s. The pure PVA is around the same variation of contact angle (55.3° to 40.67° in 150 s) close to that of the sample gelatin/PVA = 2:8. It seems that pure PVA, or PVA with a small amount of added gelatin, can sustain longer water dissolution as compared to the sample with a higher content of gelatin [53,54,55,56,57].

### 4.3. Cell Culture

**Figure 11:**
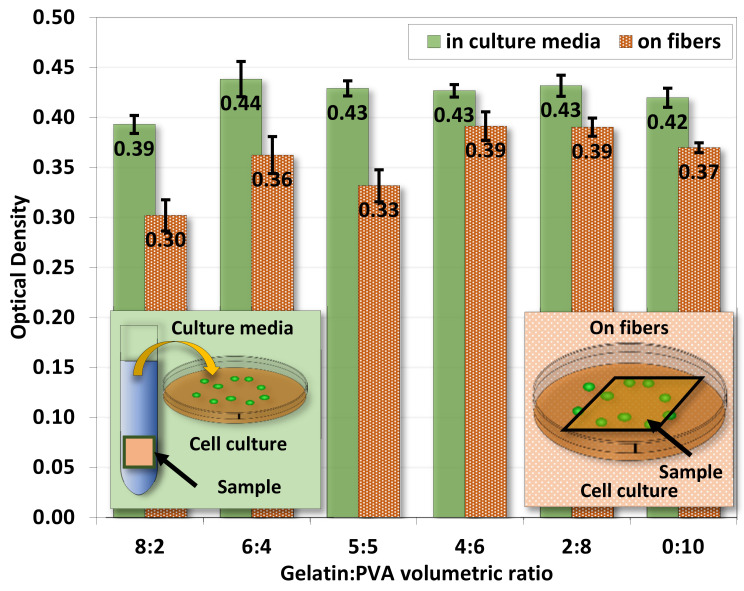
The average optical density from MTT assay for 3T3 cultured in culture media and on gelatin/PVA nanofiber films. The optical density is $\log_{10}\left[\frac{\mathrm{incident}\;\mathrm{light}\;\mathrm{intensity}}{\mathrm{transmitted}\;\mathrm{light}\;\mathrm{intensity}}\right]$.

Figure 11 shows the ratio of optical densities from the MTT assay 48 h after 3T3 seeding in sample-immersed media and on fiber film samples. Both cases show comparable optical density in each sample whether 3T3 was cultured inside sample-immersed media or directly on samples. Overall, 3T3 directly cultured on samples has a lower optical density (cell count), and this is because cells are more likely to retain on samples. Samples of gelatin/PVA = 8:2 are slightly lower than others. This result provides evidence of non-toxic chemicals released from the gelatin/PVA fiber films from the 3T3 cell culture.

**Figure 12:**
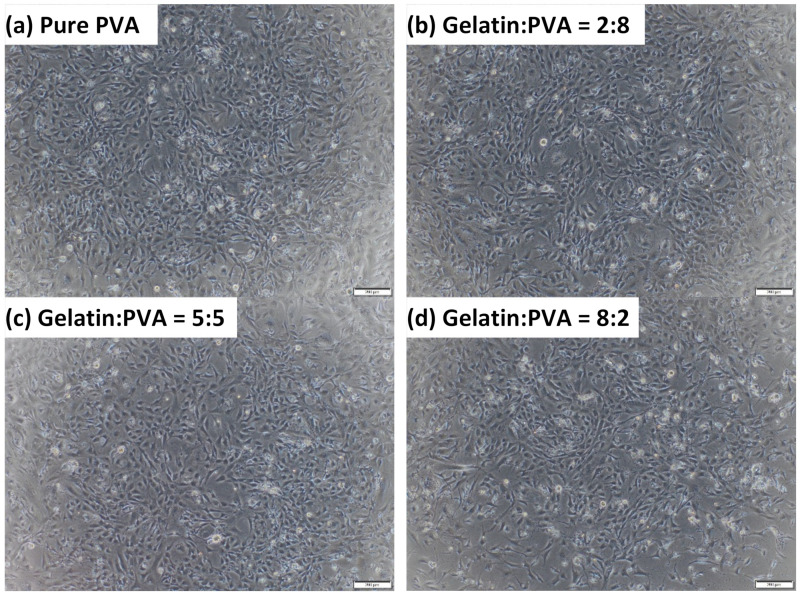
Selected optical images (50×) of cultured 3T3 in media from immersed gelatin/PVA = 8:2, 5:5, 2:8, and pure PVA.

The morphologies of 3T3 at 48 h after seeding by an optical microscope are shown in Figure 12, where images are 3T3 in 24-well plates for fiber films of gelatin/PVA = 8:2, 5:5, 2:8, and 0:10 (pure PVA). We notice that although the optical density of the MTT assay for samples of gelatin/PVA = 8:2 is slightly lower, the morphologies of 3T3 present little difference from those cultured on other fiber films. Mostly, 3T3 cells in all cases can stretch and spread as in normal connective tissues ready for migration. This is an important indication of the non-toxic micro-environment in culture media derived from the gelatin/PVA fiber films.

## 5. Discussion

### 5.1. Summary of Process Parameters for Electrospinning Nanofibers

Measurements on properties of gelatin/PVA polymeric solutions are meant to be an informative vindication for the possible formation of nanofibers by electrospinning. The regression models in Figure 2, Figure 3, Figure 4 and Figure 5 provide estimates on the relationship between volumetric ratios of gelatin/PVA mixture and viscosity, electrical conductance, pH values, and surface tension, respectively. One simple way to examine the influence of different properties on the formation of nanofibers is to inspect the slope of regression functions. With the high fidelity of these fitting functions, i.e., all coefficients of determination R^2^ are greater than 0.95 (fitting model can explain or predict 95% of the variation of data) and narrow 95% confidence intervals of those coefficients in the fitting functions, the slope of the fitting function can represent the sensitivity of properties to different concentrations of gelatin/PVA. Table 3 compares sensitivities among all properties based on their regression functions. The most sensitive property to the varying concentrations of gelatin/PVA is found to be viscosity with a slope at least three orders larger than all other properties. The second one is the surface tension but with a negative slope. Both electrical conductance and pH value are only minimally affected by the concentrations of gelatin/PVA. Thus, the statistical inference from the data indicates that the formation of nanofibers by electrospinning is largely determined by the viscosity of the polymeric solution and surface tension, which suits the mechanisms of electrospinning [58,59,60,61]. It is particularly instructive for the case of pure PVA, whose relatively lower surface eases the stretching by the electrical field in electrospinning, but higher viscosity prevents overstretching beyond the Plateau–Rayleigh instability to become droplets.

### 5.2. Fitting Model of Viscosity

Since viscosity is a dominant factor in the formation of nanofibers by electrospinning, a brief discussion on the viscosity is brought up herein. Since our polymeric solution is a mixture of two polymers, a blending of two viscosities should be expectable. Following the Lederer–Roegiers model for a binary mixture [62], the

(3) $$\ln\left(\mu_{\mathrm{mix}}\right)=\frac{x_{\mathrm{gelatin}}}{x_{\mathrm{gelatin}}+\alpha x_{\mathrm{PVA}}}\ln\left(\mu_{\mathrm{gelatin}}\right)+\frac{\alpha x_{\mathrm{PVA}}}{x_{\mathrm{gelatin}}+\alpha x_{\mathrm{PVA}}}\ln\left(\mu_{\mathrm{PVA}}\right)$$

where *μ*_●_ and *x*_●_ are, respectively, the viscosity and molar fraction of either polymer. α is an empirical constant.

One rough justification for these measurements is that when a solution has a viscosity higher than that of water its surface tension can be no higher than that of water (0.07275 J/m^2^). This is because higher surface tension is caused by intermolecular forces pulling surface molecules inwards, by which a local concentration gradient is created. This concentration gradient leads to a surface tension gradient which acts opposite to the movement. The interfacial movement is therefore damped.

Here is a reminder of earlier experimental results that the appropriate balance between higher viscosity (~420–~4300×10^−2^ poise) and slightly lower surface tension (~35.12–~32.68 mN/m^2^) of the mixed polymer solution would be favorable for the formation of nanofibers by our electrospinning system.

### 5.3. Estimation of the Interfacial Energy by Water Contact Test

**Figure 13:**
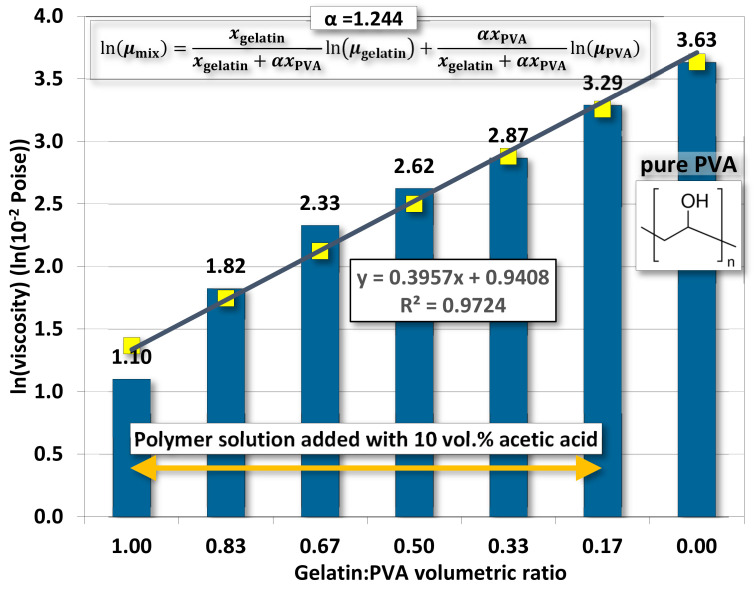
A mixture model for fitting the viscosity of gelatin/PVA mixed solutions. The empirical coefficient α is obtained by minimizing errors in the L^2^ norm (square root of the sum of error^2^) between experimental data and fitting numbers.

The wettability by water contact in Figure 10 exhibits the spreading of water drops on the surface of nanofibers. A theoretical study on the spreading contact of a drop on a plane by M. Härth and D. W. Schubert provides an insightful view of the physics of an axisymmetric drop on partially and completely wetted substrates [63]. Considering a drop on the substrate as shown in Figure 13 the spreading of the drop is controlled by the balance among three forces, namely the capillary, gravity, and viscous friction on the interface between the drop and substrate, that is,

(4) $$\underset{\mathrm{Capillary}\;\mathrm{Force}}{\underbrace{2\pi r\left(\Delta E+\gamma_{L}\frac{8V^{2}}{\pi^{2}r^{6}}\right)}}+\underset{\mathrm{Gravitational}\;\mathrm{Force}}{\underbrace{\frac{4\rho gV^{2}}{3\pi r^{3}}}}-\underset{\mathrm{Viscous}\;\mathrm{Friction}\;\mathrm{Force}}{\underbrace{\frac{\eta \dot{r}r^{6}}{\lambda^{\prime }V^{2}}}}=0$$

where *r*, *ρ*, *V*, *η*, and *γ_L_* are, respectively, the radius of the water contact circle on the substrate, the density, the total volume, the viscosity, and the surface tension (energy) of drop; *g* is the gravity constant; 2*πλ′* ≈ (37.1 ± 14.1) m^−1^ is a wetting-independent shape factor; *s* is the spreading parameter (penetration or seeping) as shown in Figure 13, and $\dot{r}=\frac{dr}{dt}$ is the rate of spreading. The last remaining variable is the interfacial energy Δ*E*,

(5) $$\Delta E=\gamma_{S}-\gamma_{L}-\gamma_{SL}$$

where *γ* is the surface energy (tension) with a subscript denoted for solid (*S*), liquid (*L*), or solid–liquid interface (*SL*). This is the unknown parameter subjected to the estimation discussed herein. Except for the surface energy of water surface energies *γ_L_*, both *γ_S_* (surface energy of nanofibers) and *γ_SL_* (interfacial energy between nanofibers and water) are unknown. *γ_S_* may be roughly estimated from the solid PVA and gelatin, but faithful values of *γ_SL_* are unfound in the literature. In fact, *γ_L_* and *γ_S_* have their unique values because of the dependence on their respect morphologies and chemical compositions.

**Figure 14:**
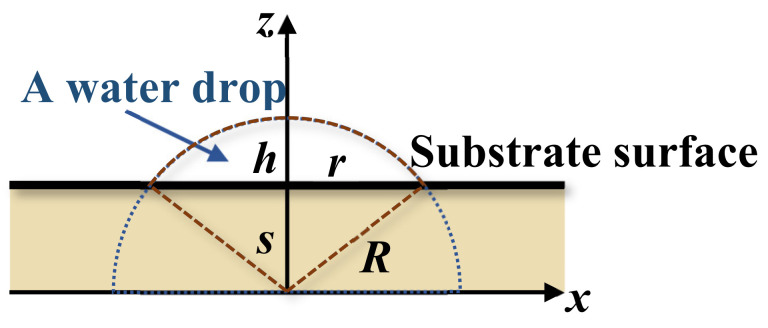
A schematic drawing of a drop of liquid on a partially wetted substrate.

Experimentally, *r* and *h* can be measured from the image of the drop. Then, the radius *R* and spreading parameter s of a partially wetting drop are determined by the following geometric relation (cf. Figure 14):

*s* + *h* = *R* (6)

*r*^2^*+ s*^2^ = *R*^2^ (7)

Therefore,

(8) $$R=\frac{h}{2}\left({\left(\frac{r}{h}\right)}^{2}+1\right)$$

(9) $$s=\frac{h}{2}\left({\left(\frac{r}{h}\right)}^{2}-1\right)\;$$

To solve equation (4) we first define a new variable,

*u* = *r*^6^ (10)

Dividing (4) by 2*πr* on both sides, we then have

(11) $$\frac{\eta \dot{r}r^{5}}{2\pi \lambda V^{2}}=\left(\Delta E+\gamma_{L}\frac{8V^{2}}{\pi^{2}r^{6}}\right)+\frac{2\rho gV^{2}}{3\pi^{2}r^{4}}$$

where *λ* = 2*πλ′* ≈ (37.1 ± 14.1) m^−1^ [63]. The initial condition for this non-linear ordinary differential equation is

*u*(0) = *r*^6^(0) (12)

This condition represents the initial spherical drop in contact with the substrate. Practically, in most experiments of contact angle cases, the initial condition should start with a finite contact width *r*_0_; therefore, the initial condition is

*u*(0) = (*r*(0))^6^ = (*r*_0_)^6^ (13)

**Figure 15:**
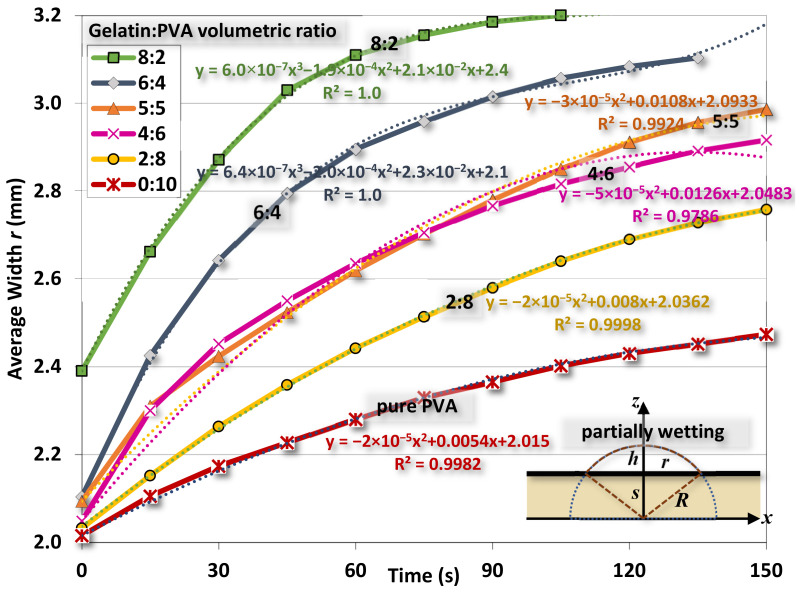
Variations of the width *r* of water contact as functions of time.

We used this condition for the following numerical analysis. The steps to determine the surface energy ∆*E* are:

- We first determine *r* and *h* at the selected time (including initial values of *r*_0_ and *h*_0_) from the image of the water drop for the contact angle test;
- Radius of the drop *R* and penetration *s* can be calculated following Equations (8) and (9). Then, the volume of the drop can be calculated by simple integration shown in Appendix A;
- For water at 25 °C, the surface tension of *γ_L_* = 0.07275 J/m^2^, viscosity is 0.0091, and poise and density are 997 kg/m^3^;
- The rate change, $\dot{r}$, is estimated from the fitting functions, as shown in Figure 15;
- The interfacial energy Δ*E* at different times is estimated from Equation (11) as
  (14) $$\Delta E=\frac{\eta \dot{r}r^{5}}{2\pi \lambda V^{2}}-\gamma_{L}\frac{8V^{2}}{\pi^{2}r^{6}}-\frac{2\rho gV^{2}}{3\pi^{2}r^{4}}$$
- Plugging values of Δ*E* at each time back to eq. (11) to re-calculate $\dot{r}$. These $\dot{r}$ are different from the $\dot{r}$ estimated from the fitting functions;
- Among various values of Δ*E* at each time, an optimal value that minimizes the difference of $\dot{r}$ between the fitting function and the previous step can be determined accordingly.

**Figure 16:**
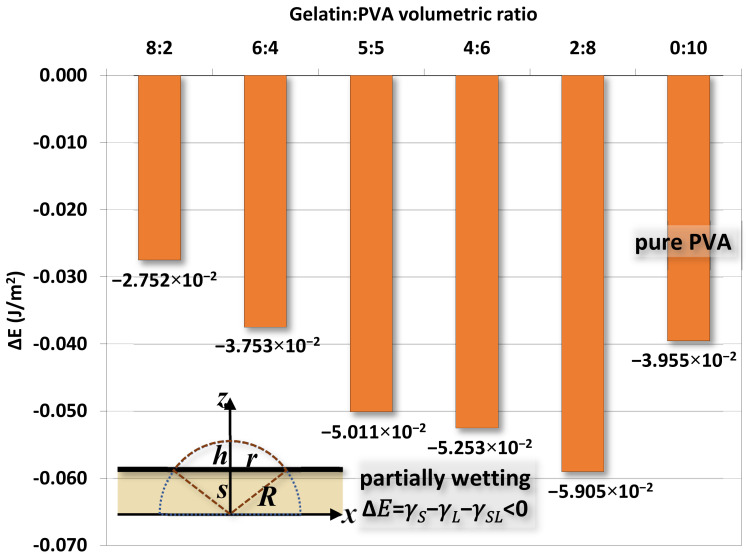
The optimal estimate of interfacial energy ∆*E* from the width *r* of water contact and model proposed by Härth and Schubert.

Figure 15 shows the variation of width *r* of water drops at different times for different gelatin/PVA concentrations. As time elapsed, the drop gradually flattens as *r* increases. Using the procedure just mentioned, the estimated interfacial energies ∆*E* for different compositions of nanofibers are shown in Figure 16. The optimal estimate of ∆*E* in the range between ~−0.028 and ~−0.059 J/m^2^ is found for different nanofibers. The negative values of ∆*E* confirm the partial wetting regime as indicated in [63]. If considering the surface energy of pure water at 25 °C and *γ_L_* ≈ 0.72 J/m^2^, the pure gelatin or pure PVA *γ_S_* ≈ 0.033–0.29 J/m^2^ [64,65,66] and then the interfacial *γ_SL_* = (*γ_S_* − *γ_L_* − ∆*E*) would be approximately (−0.0259–0.2311 J/m^2^) and (−0.0146–0.2424 J/m^2^). The negative values are physically infeasible as the interface between nanofibers and water becomes unstable. Since there is a lack of data in the literature, these values are referential for further investigations.

## 6. Conclusions

In this study, we investigated the relationship between the compositions of gelatin/PVA mixed hydrogels and the electrospinning process. The electrospun gelatin nanofibers mixed with different concentrations of PVA were deposited on a layer film of the same compositions by spin coating. An important point is to determine a window of composition for the successful fabrication of nanofibers. The investigation was conducted twofold. One front was to measure the properties of polymer solutions including viscosity, pH value, electrical conductance, and surface tension. Another side was to analyze the compositions and properties of nanofibers by FTIR, SEM, and EDS. For the polymer solutions, we found that viscosity is a dominant factor influencing the formation of nanofibers, and surface tension is a secondary factor. The pH value and electrical conductance are only marginal factors. The FTIR confirms that gelatin and PVA are physically miscible during the formation of nanofibers with the support of surface elemental analysis by EDS and SEM images of nanofibers. The hydrophilicity of nanofibers was tested by water contact angle at a prolonged period. As time expands, water drops gradually flattened as an indication of becoming more hydrophilic. We specifically introduced a theoretical model by Härth and Schubert for the spread of water contact to estimate the interfacial surface energy between the water drop and nanofibers. A range of −0.02752–0.2763 J/m^2^ was predicted for the mixed gelatin/PVA nanofibers. In addition to the material characterizations, specially chosen gelatin/PVA nanofiber samples were tested for their biotoxicity via the cell culture of 3T3 fibroblasts. Both optical images and MTT assays show that 3T3 spreads and proliferates healthily after 48 h of seeding without signs of necrosis.

## Figures and Tables

**Table 1:** Parameters for spin coating of gelatin/PVA mixed layer film.

Process Parameters for Gelatin/PVA Films by Spin Coating	
Speed (rpm)	3000
Operational temperature (°C)	Room temperature
Deposition time (second)	60
Gelatin concentration in DI water (wt. %)	12
PVA concentration in DI water (wt. %)	12
Acetic acid concentration in DI water (vol. %)	10 mL/100 mL (10%)
Mixture of polymeric solution volume ratio (vol. %)	8:2, 6:4, 5:5, 4:6, 2:8, 0:10 in total 10 mL

**Table 2:** Parameters for electrospinning gelatin/PVA mixed nanofibers.

Process Parameters for Gelatin/PVA Nanofibers by Electrospinning	
Voltage (kV)	12–12.5
Syringe pumping speed (μL/min)	1 × 10^−2^
Syringe outer/inner diameter (mm)	5.0 × 10^−1^/2.6 × 10^−1^
Operational temperature (°C)	Room temperature
Working distance (cm)	12
Deposition time (sec)	>60

**Table 3:** Statistical information of the fitting functions for the measurements of viscosity, electrical conductance, pH values, and surface tension of different gelatin/PVA mixed solutions.

	Volumetric Ratio Gel:PVA						
	10:0	8:2	6:4	5:5	4:6	2:8	0:10
**Viscosity**							
8.7258×e^0.91109x^							
**Coefficient of Determination R^2^**	0.972						
**95% confidence interval**	6.2662 ≤ 8.7258 ≤ 12.1508 0.8370 ≤ 0.91109 ≤ 0.9851						
**Sensitivity (slope of fitting function)**	19.7542	49.1432	122.2549	304.1370	756.6103	1882.2410	4682.5044
**pH**							
y = 5.5143×10^−2^x + 3.232							
**Coefficient of Determination R^2^**	0.983						
**95% confidence interval**	3.2088 ≤ 3.232 ≤ 3.2552 0.0492 ≤ 0.055143 ≤ 0.0611						
**Sensitivity (slope of fitting function)**	0.0551						
**Electrical Conductance**							
y = −3.6419×10^−4^x + 2.7648×10^−3^							
**Coefficient of Determination R^2^**	0.966						
**95% confidence interval**	0.0025 ≤ 0.0027648 ≤ 0.0030 −0.0003 ≤ −0.00036419 ≤ −0.0004						
**Sensitivity (slope of fitting function)**	−3.6419×10^−4^						
**Surface Tension**							
y = −1.0827x + 39.973							
**Coefficient of Determination R^2^**	0.982						
**95% confidence interval**	39.2904 ≤ 39.973 ≤ 40.6562 −1.2354 ≤ −1.0827 ≤ −0.9300						
**Sensitivity (slope of fitting function)**	−1.0827						

## Data Availability

The data presented in this study are available on request from the corresponding author.

## Appendix A. Integration of Drop Volume in the Contact Angle Test

The volume of the water drop in the contact angle test can be calculated by simple integration. Assuming axisymmetry of the drop along the z-axis in Figure 12, the drop volume *V* is

(A1) $$\int_{s}^{R}\pi r^{2}dz=\int_{s}^{R}\pi \left(R^{2}-z^{2}\right)dz=\pi \left(\frac{2}{3}R^{3}-\left(R^{2}s\right)-\frac{1}{3}s^{3}\right)$$

## Appendix B. Major FTIR Absorption Peaks

Some FTIR absorption peaks are listed in Table A1 for readers’ reference.

**Table A1:** Major FTIR absorption peaks for gelatin and PVA [40,41,42,43,44,45,46,47,48,49,50].

Wavenumber (cm^−1^)	Gelatin Mode	Reference
400–900	–CO–NH–moiety, amide IV-VI bands	[41]
670–1240	Amide III (C-N, in-plane bending vibrations of N-H, weak C-C bond, and C=O)	[43]
900–1900	Amide I, II, and III	[41]
1238	Delta N-H, C-N stretching	[40]
1245	Amide III	[44]
1300–1560	Amide II (N-H bond-bending mode and stretching vibration of C-N bond)	[43]
1328	Wagging vibration of proline side chains	[42]
1400	Symmetric O–C–O stretching	[40]
1500–1550	N-H deformation	[42]
1540	Amide II	[42]
1541	Delta N-H, C-N stretching	[40]
1560	Amide II	[44]
1620–1660	Amide I (stretching of carbonyl C=O (peptide bond) with less involvement of the stretching C-N bond)	[43]
1637	C=O stretching	[42]
1650	Amide I	[44]
1653	C=O, CN stretching	[40]
2300–3600	Amide A	[43]
2700–3600	Amide A and B	[41]
2947	C-H stretching	[42]
2960–2935	Asymmetric and symmetric CH_2_ stretching	[40]
3065	N-H stretching	[40]
3270–3370	N-H stretching	[42]
3306	N-H, O-H stretching	[40]
**Wavenumber (cm^−1^)**	PVA Mode	Reference
839	C-C stretching vibration	[48]
849	C-H rocking mode	[50]
1000–1100	C–O stretching in C–O–H groups and COC groups	[50]
1081	C–O stretching of acetyl groups	[48]
1085–1150	C–O–C	[47]
1100	C-O-C	[45]
1140	Crystalline C–O stretching	[50]
1141	C–O (crystallinity)	[47]
1248	C–O stretching	[50]
1261	C–O stretching	[50]
1324	C–H deformation vibration	[48]
1377	CH_3_ bending	[45]
1417–1461	Delta CH_2_	[47]
1425	C–H bending vibration of CH_2_	[48]
1435	C–H bending	[50]
1440	CH_2_ bending	[45]
1680–1730	C=O and C-O stretch from the remaining acetate groups	[46]
1690	C=O carbonyl stretch	[48]
1720–1737	C=O stretching	[45]
1732	C=O stretching	[50]
1735–1750	C=O	[47]
2840	Symmetric stretching vibrational of C–H from alkyl groups	[49]
2840–3000	C-H stretch from alkyl groups	[46]
2914	C–H stretching from the alkyl groups	[50]
2917	CH_2_ asymmetric stretching	[48]
2920	Antisymmetric stretching vibrational of C–H from alkyl groups	[49]
2929	CH sp3	[45]
2943	C–H stretching from the alkyl groups	[50]
3200–3550	O-H stretching	[46]
3280	O–H stretching	[48]
3369	O–H stretching	[50]
3400	O–H stretching	[49]
3411	O-H stretching	[45]
